# Cotranslational protein-RNA associations predict protein-protein interactions

**DOI:** 10.1186/1471-2164-15-298

**Published:** 2014-04-22

**Authors:** Caia DS Duncan, Juan Mata

**Affiliations:** 1 Department of Biochemistry, University of Cambridge, Cambridge, UK

## Abstract

**Background:**

Most cellular proteins function as part of stable protein complexes. We recently showed that around 38% of proteins associate with mRNAs that encode interacting proteins, reflecting the cotranslational formation of the complex between the bait protein and the nascent peptides encoded by the interacting mRNAs. Here we hypothesise that these cotranslational protein-mRNA associations can be used to predict protein-protein interactions.

**Results:**

We found that the fission yeast Exo2 protein, which encodes an exonuclease of the XRN1 family, coimmunoprecipitates with the *eti1* mRNA, which codes for a protein of unknown function and uninformative sequence. Based on this protein-mRNA association, we predicted that the Exo2 and Eti1 protein are part of the same complex, and confirmed this hypothesis by coimmunoprecipitation and colocalization of the proteins. Similarly, we show that the cotranslational interaction between the Sty1 MAP kinase and the *cip2* mRNA, which encodes an RNA-binding protein, predicts a complex between Sty1 and Cip2.

**Conclusions:**

Our results demonstrate that cotranslational protein-mRNA associations can be used to identify new components of protein complexes.

## Background

**Figure 1:**
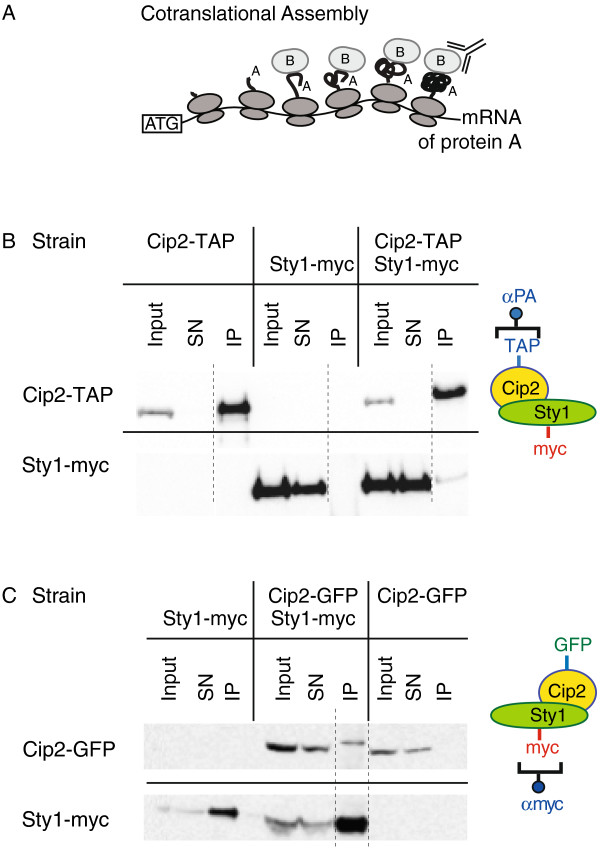
**The Cip2 and Sty1 proteins associate with each other. (A)** Cotranslational assembly and its detection by RIP-chip. Protein B binds to the nascent peptide of protein A. When protein B is immunoprecipitated, it copurifies with protein A and its corresponding polysome. Protein B is identified by its cognate mRNA. **(B)** Cells expressing Cip2-TAP, Sty1-myc, or both were used for immunoprecipitation experiments with antibodies against protein A. Samples were analysed by Western blotting and probed with peroxidase anti-peroxidase complexes to detect the TAP tag (top panel), or with antibodies against the myc epitope (bottom panel). Equivalent amounts of proteins were loaded for the cell extract (Input) and the supernatant after purification (SN), and the immunoprecipitate (IP) was concentrated at least 5-fold with respect to the original extracts. All samples were run on the same gel. The dotted line indicates where a lane was removed from the image. Note that Sty1-myc is only detected in the IP when Cip2-TAP is coexpressed. **(C)** As in B, but cells expressed Cip2-GFP, Sty1-myc or both. Cip2-GFP is only detected in the IP when Sty1-myc is coexpressed.

Although most cellular proteins are part of protein complexes, little is known about how the formation of protein complexes occurs *in vivo*. To address this question we recently used a method called RIP-chip (Ribonucleoprotein ImmunoPrecipitation analysed with DNA chips) to identify mRNAs associated with specific proteins. We found that ~38% of a set of proteins with varied structures and functions interacted with mRNAs known or predicted to encode proteins that interact with the bait proteins. We also discovered that these associations required the presence of the protein encoded by the mRNA as well as active translation. These results indicated that the interaction between the bait protein and the protein encoded by the associated mRNA takes place cotranslationally (i.e., as the second protein is being translated). The immunoprecipitated protein is associated with the nascent peptide together with the mRNA that is being translated, allowing the identification of the nascent peptide through its mRNA (Figure 1A). This appeared to be a widespread phenomenon, and applied to proteins with diverse structures and function, such as protein kinases, kinesins, proteasome components, transcription factors, etc. [1]. Similar results had been obtained for individual protein-mRNA pairs [2-6], but our work and that of others demonstrated that this phenomenon could be addressed at the genome-wide level and in an unbiased manner using RIP-chip [1,7,8]. Our observations suggested that protein-mRNA interactions discovered by RIP-chip could be used to identify novel protein-protein interactions. To test this hypothesis we followed up a previously described interaction between the Sty1 MAP-kinase and the *cip2* mRNA [1], which encodes an RNA-binding protein, and found that both proteins coimmunoprecipitate. Moreover, we present a novel interaction between the Exo2 protein (an XRN1-family exonuclease) and an mRNA that encodes an uncharacterised protein that shows no homology to other proteins (*eti1*). As hypothesised, we found that the two proteins coimmunoprecipitate *in vitro* and colocalise *in vivo*. This work demonstrates that genome-wide protein-mRNA interaction datasets can be used to predict protein-protein associations.

## Results and discussion

The Sty1 protein is a MAP kinase that mediates stress responses in fission yeast. RIP-chip experiments revealed that Sty1 interacts with two mRNAs [1]: *pyp2*, which encodes a protein phosphatase known to physically interact with Sty1 [9], and *cip2*, which codes for an RNA-binding protein that regulates the response to oxidative stress [10]. We have shown that the interaction between the Sty1 protein and the *cip2* mRNA requires active translation of *cip2*, as it is disrupted when translation is inhibited using puromycin and when the *cip2* initiation codon is mutated [1]. This strongly suggested that Sty1 binds to Cip2 as the Cip2 protein is being synthesised on the polysome. We therefore predicted that the Sty1 and Cip2 proteins interact with each other. To test this hypothesis we epitope-tagged Sty1 and Cip2, and used strains carrying the tagged proteins for coimmunoprecipitation experiments. As predicted, Sty1-myc copurified specifically with Cip2-TAP (Figure 1B). To confirm the specificity of the interactions we repeated the experiment using Sty1-myc and Cip2-GFP. Consistent with the previous result, Cip2-GFP copurified with immunoprecipitated Sty1-myc (Figure 1C). Moreover, the interaction Cip2-TAP and Sty1-myc was not disrupted by treatment with RNase, indicating that the associations are not mediated by the *cip2* mRNA, and thus that both proteins are part of the same complex (Additional file 1: Figure S1A). These results show that RIP-chip data can be used to predict protein-protein interactions.

**Figure 2:**
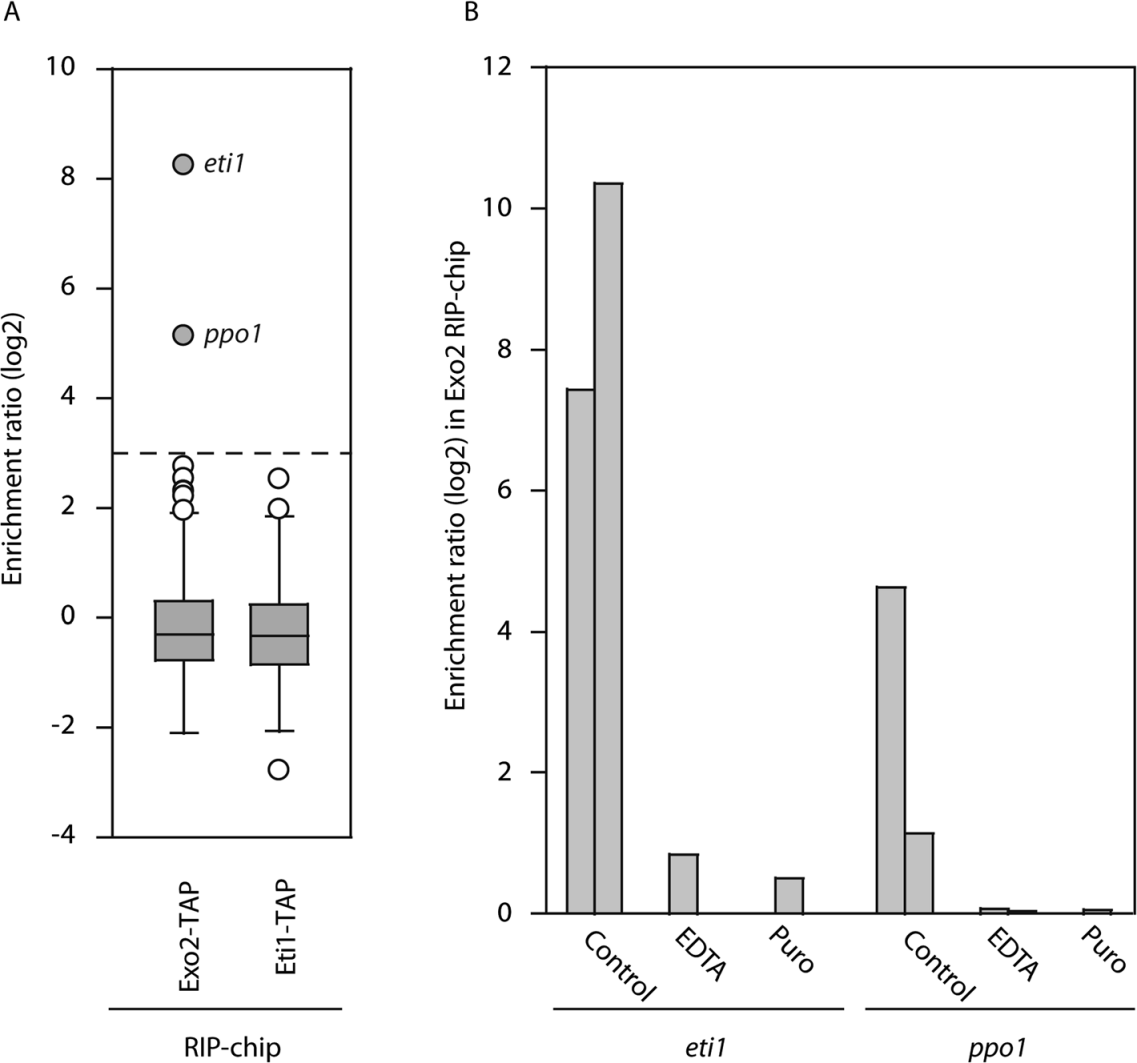
**RIP-chip experiments with Exo2 and Eti1 proteins. (A)** Box plots of the distribution of enrichments for RIP-chip experiments with Exo2 and Eti1. The y axis shows normalised log_2_ enrichment ratios in the indicated RIP-chip experiments. White circles represent mRNAs that were not reproducibly enriched, while grey circles correspond to transcripts that consistently associated with the proteins. Exo2 interacts with only two mRNAs (*eti1* and *ppo1*), while Eti1 does not associate with any transcripts. **(B)** Enrichment levels of *eti1* and *ppo1* mRNAs in Exo2 RIP-chips after cells were treated with puromycin or extracts were incubated with EDTA. The results for two independent biological replicates are shown. Both treatments disrupt the interactions between Exo2 and the transcripts, indicating that the interactions are cotranslational.

**Figure 3:**
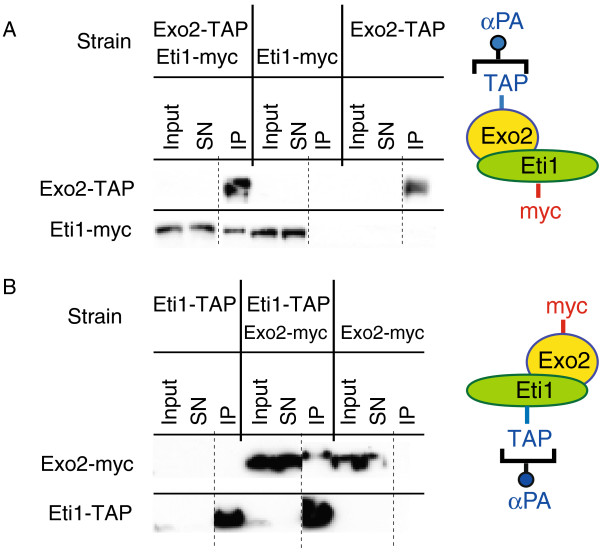
**The Exo2 and Eti1 proteins interact with each other. (A)** Cells expressing Exo2-TAP, Eti1-myc, or both, were used for immunoprecipitation experiments with antibodies against protein A. Samples were analysed by Western blotting and probed with peroxidase anti-peroxidase complexes to detect the TAP tag (top panel), or with antibodies against the myc epitope (bottom panel). Equivalent amounts of proteins were loaded for the cell extract (Input) and the supernatant after purification (SN), and the immunoprecipitate (IP) was concentrated at least 5-fold with respect to the original extracts. All samples were run on the same gel. The dotted line indicates where a lane was removed from the image. Eti1-myc is only detected in the IP when Exo2-TAP is coexpressed. **(B)** As in **A**, but cells expressed Exo2-myc, Eti1-Tap, or both. Exo2-myc is specifically associated with Eti1-TAP. Note that myc-tagged proteins are detected more efficiently than TAP-tagged versions (probably due to the presence of 13 copies of the myc epitope). This explains why Exo2-myc, but not Exo2-TAP, can be detected in cell extracts (cf. panels **A** and **B**).

To confirm the general applicability of this principle, we decided to use a binding partner of unknown function. As part of a study of the regulation of mRNA decay in fission yeast, we performed RIP-chip experiments with the Exo2 protein, which is the *S. pombe* homologue of the XRN1 5′- > 3′ exonuclease [11] (Figure 2A). A TAP-tagged version of Exo2 copurified with only two mRNAs: *SPBC19G7.10c*, and *SPAC12G12.09. SPBC19G7.10c* encodes an orthologue of the PAT1 protein, which is a known interactor of XRN1 in yeast and higher eukaryotes [12], and we refer to it hereafter as *ppo1* (for *pombe pat one*; note that there is already a gene called *pat1* in *S. pombe*). *SPAC12G12.09* encodes a predicted protein of 977 amino acids that lacks any known domain and does not display homology to any other protein. We named this gene *eti1*, for *exo two interacting* (see below). To confirm that these interactions reflected cotranslational assembly, we incubated cells expressing Exo2-TAP with puromycin to disrupt active translation, and used them for RIp-chip experiments. This treatment led to a complete loss of interaction between Exo2 and both *ppo1* and *eti1* mRNAs (Figure 2B). Moreover, disruption of polysomes *in vitro* by incubating Exo2-TAP extracts with EDTA caused a similar effect (Figure 2B). We also tested if these interactions were symmetrical, that is, whether Eti1 would interact with the *exo2* and *ppo1* mRNAs. RIp-chip experiments with Eti1-TAP (Figure 2A) did not reveal any enriched mRNAs, indicating that the interactions were unidirectional. This might indicate that Exo2 can only bind to Eti1 *in vivo* before Eti1 is fully translated and folded. Based on these data, we hypothesised that the Exo2 and Eti1 proteins form a complex *in vivo*. To test this idea we made strains expressing TAP-tagged Exo2 and myc-tagged Eti1. myc-tagged Eti1 coprecipitated with TAP-tagged Exo2, indicating that both proteins interact (Figure 3A). A strain containing Eti1-TAP and Exo2-myc showed a similar association (Figure 3B), confirming that the interactions were not dependent on the specific epitope tags used for the experiment. Furthermore, the interactions were resistant to RNase treatment, indicating that both proteins are part of the same complex (Additional file 1: Figure S1B).

To prove that the association also takes place *in vivo*, we tagged Exo2 and Eti1 with different fluorescent proteins. Exo2 and Eti1 could be detected by fluorescence microscopy in very weak cytoplasmic foci reminiscent of Processing bodies (PBs) in vegetatively growing cells (Additional file 1: Figure S2A). PBs are large cytoplasmic RNA – protein complexes that include multiple proteins involved in mRNA degradation [13], such as components of the decapping complex (Dcp1 and Dcp2) and the 5′- > 3′ exonuclease Exo2 [14]. However, PBs do not contain the poly(A) binding protein Pabp [15]. To investigate the identity of these granules we coexpressed Exo2 and Eti1 with the PB marker Dcp2. As previously reported [14], we could observe Dcp2 in discrete but weak cytoplasmic foci (Additional file 1: Figure S2A). Although the weakness of the fluorescence signals of the tagged proteins precluded us from performing extensive the colocalization studies, we detected structures containing both Dcp2 and Eti1 (Additional file 1: Figure S2B), suggesting that Eti1 may be a component of PBs.

**Figure 4:**
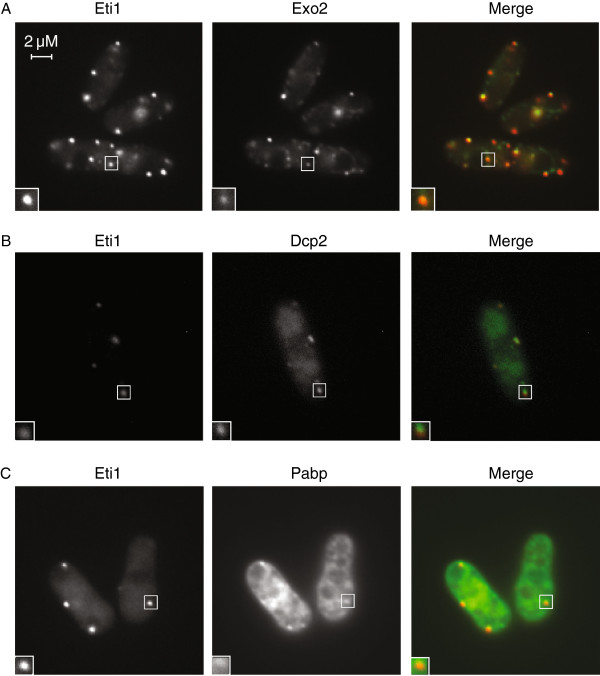
**Exo2 and Eti1 proteins colocalize in stress granules. (A)** Cells expressing Eti1-mCherry (left) and Exo2-GFP (centre) were imaged 1 hour after glucose removal. The right panel shows a merge or both images, with Eti1-mCherry in red and Exo2-GFP in green. **(B)** As **A**, but cells expressing Eti1-mCherry and Dcp2-CFP as indicated. **(C)** As **A**, but cells expressing Eti1-mCherry and Pabp-GFP as shown in the figure.

Glucose starvation caused the formation of bright cytoplasmic foci that contained both Eti1 and Exo2, supporting the biochemical data on the coprecipitation of both proteins (Figure 4A). These structures were reminiscent of stress granules, which are cytoplasmic aggregates of proteins and mRNAs formed in response to environmental stress. To characterize these foci further we used Pabp as a well-established marker of stress granules [15]. Both Eti1 and Exo2 clearly colocalized with Pabp and Dcp2, suggesting that both proteins are components of stress granules (Figure 4B-C and Additional file 1: Figure S3).

Finally, we tested whether Exo2 and Eti1 required each other for their localization. In *eti1Δ* cells, as in a wild type background, Exo2-GFP was visualized as very weak foci in vegetative cells, but moved to stress granules upon glucose starvation (Additional file 1: Figure S4A). Pabp-GFP also relocalized normally, indicating that Eti1 is not required for stress granule assembly (Additional file 1: Figure S4B). By contrast, Eti1 was present in bright granules in *exo2Δ* cells even in unstressed cells (Additional file 1: Figure S4C). Inhibition of RNA decay by inactivation of XRN1/Exo2 in yeast causes an increase in the number and size of PBs in budding yeast cells, presumably due to the accumulation of decay intermediates [13]. Eti1-containing granules in *exo2Δ* mutants did not contain Pabp (Additional file 1: Figure S4D), consistent with the idea that these foci represent PBs.

Altogether, biochemical and cytological data confirm that Exo2 and Eti1 are part of the same complex, and suggest that Eti1 is a novel component of stress granules.

## Conclusions

Our results demonstrate that cotranslational mRNA-protein interactions detected using RIP-chip can be used to predict protein-protein associations. The nature and behaviour of the proteins we identified suggest that the interactions may have biological importance. For example, there is evidence that Sty1 regulates the phosphorylation of Cip2: Cip2 is phosphorylated in response to oxidative stress, and this phosphorylation does not take place in *sty1* mutants [10]. Our results demonstrate that Sty1 and Cip2 are associated, and suggest that Sty1 may directly phosphorylate Cip2. In the case of Eti1, the interactions between Exo2 and *eti1* allowed us to identify a novel component of stress granules, which might be involved in RNA decay. Although only a small fraction of protein-protein interactions appear to form cotranslationally [1], RIP-chip experiments often identify key partners of high biological significance and have a very low false positive rate. For example, RIP-chip with the Cdc2 protein, the fission yeast CDK1, identified mRNAs encoding key regulators of the G1/S transition (*rum1*) and of S phase initiation (*cdc18*) [1]. It is important to note that the associations revealed by RIp-chip may not be direct. For example, a preformed protein complex might interact with a nascent peptide. We propose that RIP-chip can serve as a complementary method to mass spectrometry-based techniques to identify interacting partners of a given protein.

## Methods

Standard methods were used for growth and manipulation of fission yeast cells [16]. All strains used in this work are listed in Additional file 1: Table S1. All experiments were performed with cells grown in Edinburgh Minimal Medium (EMM) with the appropriate supplements at 32°C. For glucose starvation experiments, cells were incubated in EMM that did not contain glucose for 1 hour. For coimmunoprecipitation experiments cell extracts were prepared as described in [17]. Proteins were TAP-, GFP-, and myc- tagged using one-step PCR methods [18,19]. A full list of oligonucleotides is presented in Additional file 1: Table S2. TAP-tagged proteins were immunoprecipitated using monoclonal antibodies against protein A (Sigma), and detected by Western Blot using peroxidase-anti-peroxidase soluble complexes (Sigma). Myc-tagged proteins were immunoprecipitated and detected by Western blot using the 9E11 monoclonal antibody (Abcam). GFP-tagged proteins were detected with the B2 monoclonal antibody (Santa Cruz). The requirement of the interactions for intact RNA was tested by treating 250 μl of cell extract with 200 units of RNase I (Life Technologies) for 15 minutes at room temperature. For microscopy experiments, living cells expressing the indicated fusion proteins were visualized using an AxioImager M1 by Carl Zeiss MicroImaging. RIp-chip experiments, puromycin and EDTA treatments were carried out as previously described [1]. Custom-designed oligonucleotide microarrays were manufactured by Agilent, and were processed and analysed as previously described [1].

## Competing interests

The authors declare that they have no competing interests.

## Authors’ contributions

JM and CD designed the study, performed the data analysis and wrote the paper. All experiments were carried out by CD. Both authors read and approved the final manuscript.

## Supplementary Material

Additional file 1 **Microarray data: The data sets supporting the results of this article are available in the ArrayExpress repository with accession number E-MTAB-1856.** Additional supporting data includes four figures (Additional file 1: Figure S1, S2, S3 and S4) and two tables (Additional file 1: Table S1 and S2) provided as a single PDF file. Click here for file
