# Supplementary material for: Cotranslational protein-RNA associations predict protein-protein interactions
Source: BMC Genomics. 2014 Apr 22;15:298. doi: 10.1186/1471-2164-15-298 (PMC4234486; doi:10.1186/1471-2164-15-298)
Supplement: Additional file 1 — Microarray data: The data sets supporting the results of this article are available in the ArrayExpress repository with accession number E-MTAB-1856. Additional supporting data includes four figures (Additional file 1: Figure S1, S2, S3 and S4) and two tables (Additional file 1: Table S1 and S2) provided as a single PDF file. [file 1471-2164-15-298-S1.pdf]

A

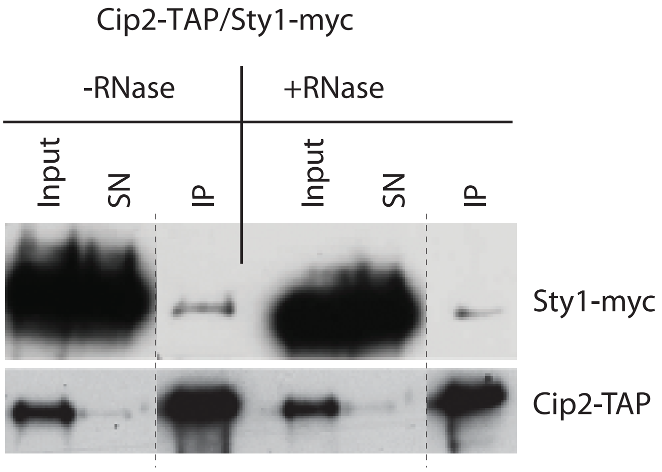

B

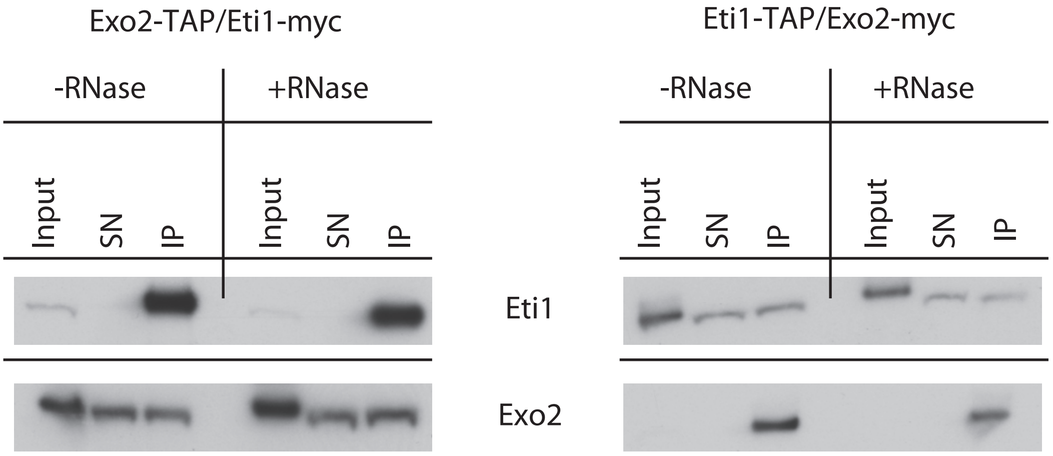

Figure S1: The interactions between Cip2 and Sty1, and between Exo2 and Eti1, do not require RNA.

(A) Cells expressing Cip2-TAP and Sty1-myc were used for immunoprecipitation experiments with antibodies against protein A. Samples were incubated with RNase I (+RNase) or mock-treated (-RNase), analysed by Western blotting, and probed with antibodies against the myc epitope or with peroxidase anti-peroxidase complexes to detect the TAP tag. Equivalent amounts of proteins were loaded for the cell extract (Input) and the supernatant after purification (SN), and the immunoprecipitate (IP) was concentrated 10 times with respect to the original extracts. All samples were run on the same gel. The dotted line indicates where a lane was removed from the image. (B) As in (A), but the experiment was performed with cells expressing Exo2-TAP and Eti1-myc, and with cells containing Eti1-TAP and Exo2-myc. All the interactions are resistant to treatment with RNase.

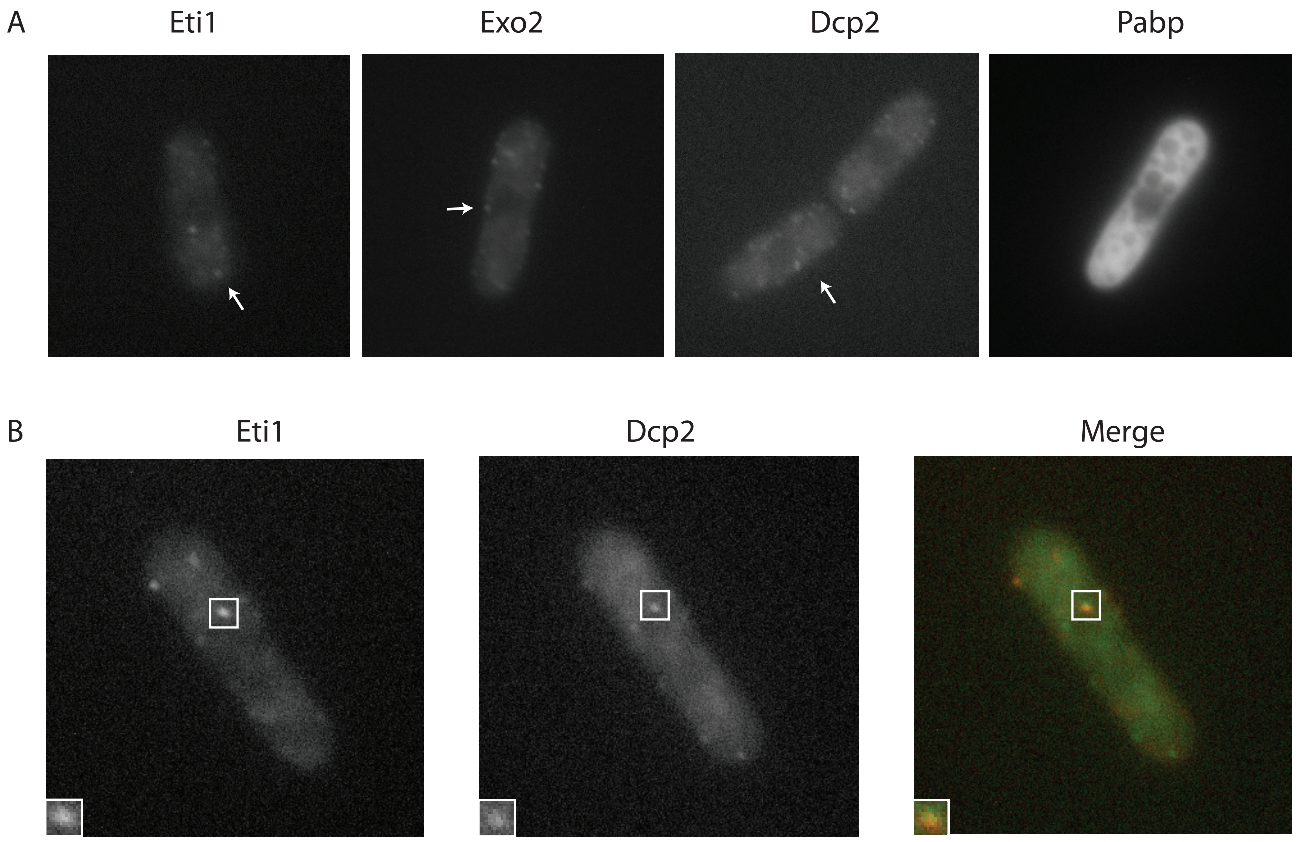

Figure S2: Localization of Eti1, P body and stress granule components in vegetatively growing cells.

(A) Vegetatively growing cells expressing Eti1-mCherry, Exo2-GFP, Dcp2-CFP, or Pabp-CFP as indicated. For all proteins except Pabp we could detect very weak cytoplasmic foci (arrows). (B) Vegetatively growing cells coexpressing Eti1-mCherry (red) and Dcp2-CFP (green).

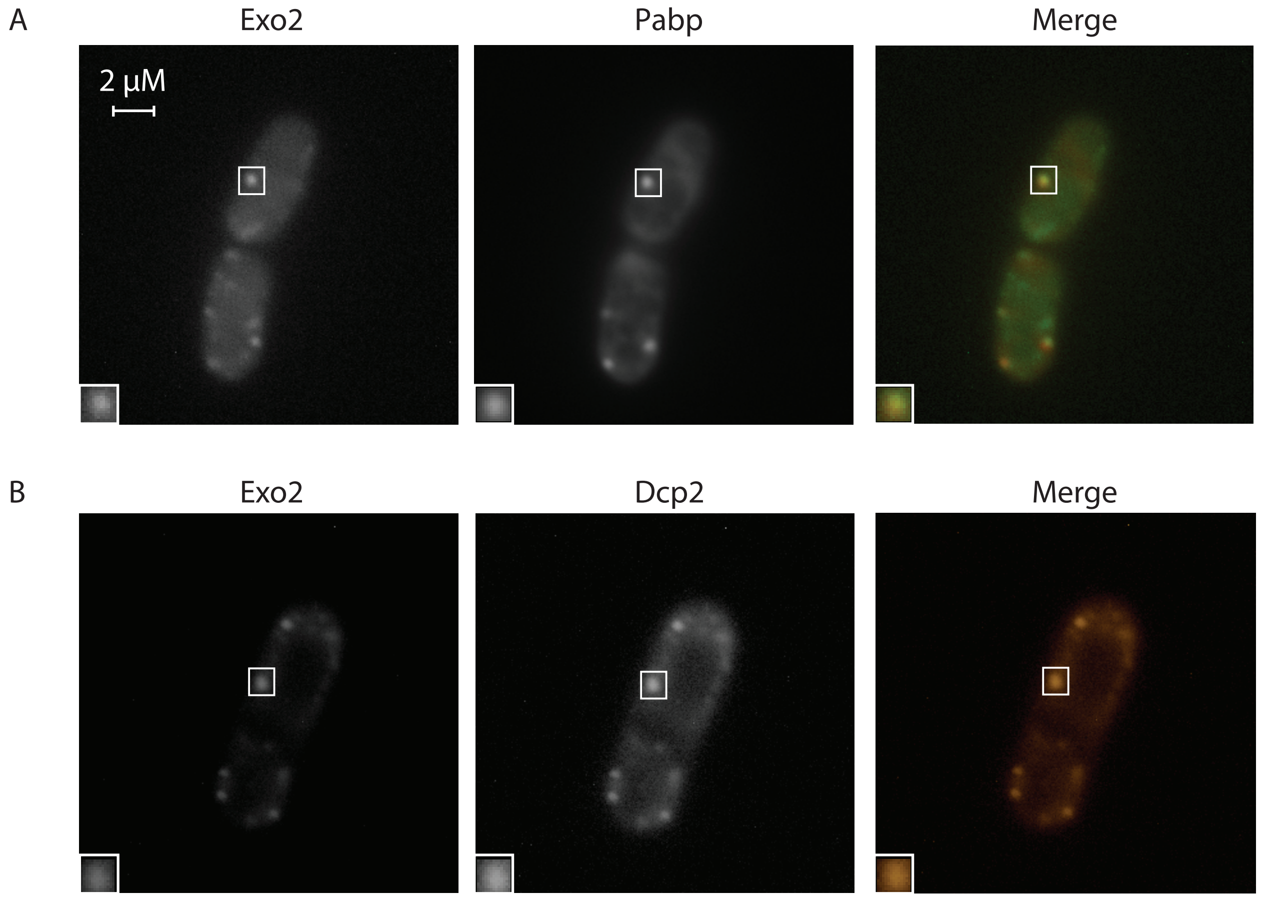

Figure S3: Exo2 colocalizes with Pabp and Dcp2 in stress granules.
(A) Cells expressing Exo2-GFP (left) and Pabp-CFP (right) were imaged 1 hour after glucose removal. The right panel shows a merge or both images, with Exo2-GFP in red and Pabp-CFP in green. (B) As A, but cells expressing Exo2-GFP and Dcp2-CFP.

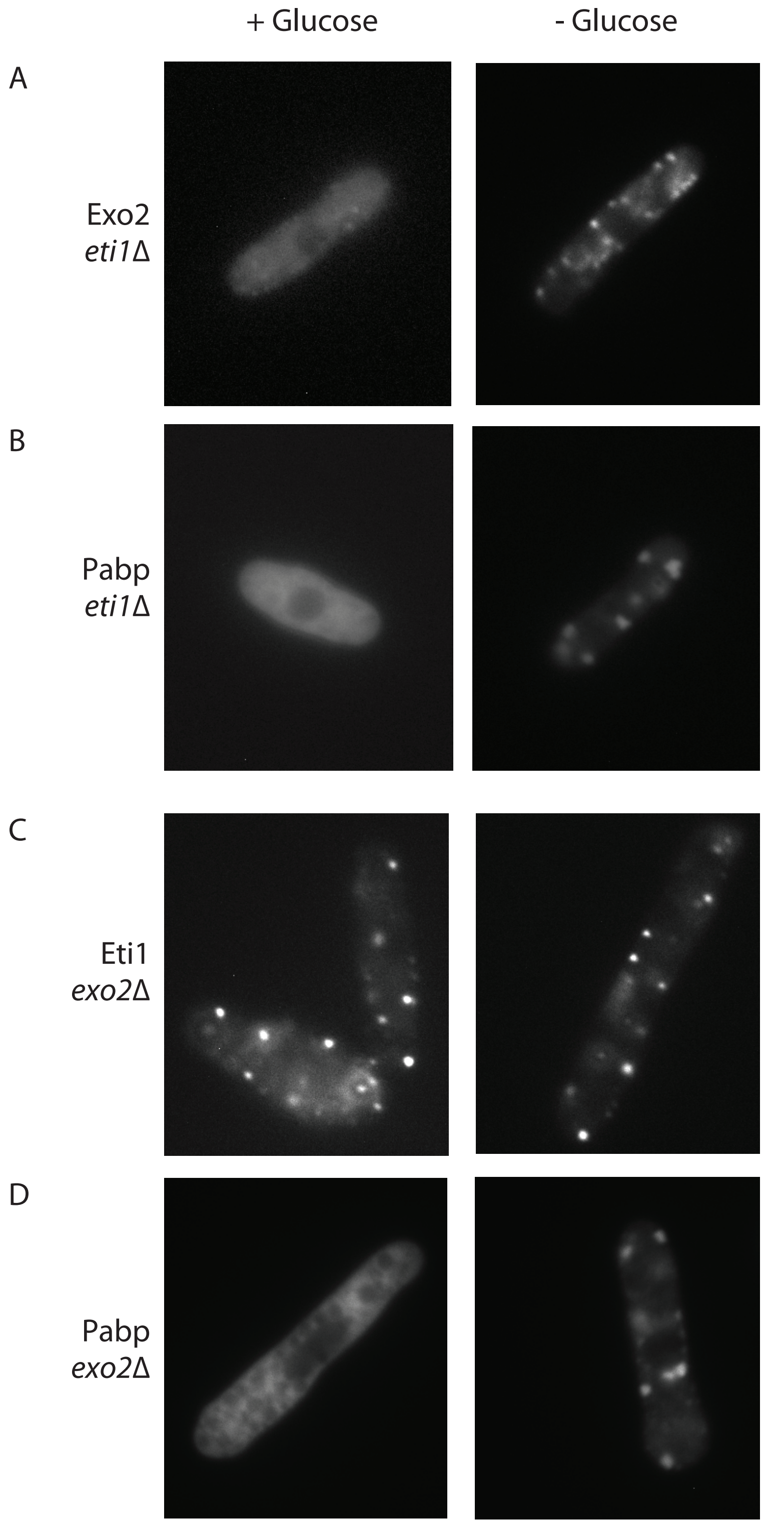

Figure S4: Dependencies between Exo2 and Eti1 for their localizations.
(A) Cells expressing Exo2-GFP in *eti1Δ* background were imaged during growth in the presence of glucose (left) and 1 hour after glucose removal. (B) As in A, but *eti1Δ* cells expressing Pabp-GFP. (C) As in A, but *exo2Δ* cells expressing Eti1-mCherry. (D) As in A, but *exo2Δ* cells expressing Pabp-GFP.

Genotype	Source
cip2-4x-TAP::natMX6 h+	This work
sty1-13x-myc::kanMX6 leu1-32 ade6-M216 h-	National BioResource Project (NBRP), Osaka, Japan
cip2-4x-TAP::natMX6 sty1-13x-myc::kanMX6 leu1-32 ade6-M216 h?	This work
exo2-2x-TAP::kanMX6 h90	This work
eti1-2x-TAP::kanMX6 h+	This work
exo2-13x-myc::natMX6 h-	This work
eti1-13x-myc::natMX6 ade6- h?	This work
eti1-13x-myc::natMX6 exo2-2x-TAP::kanMX6 ade6-M216 h?	This work
eti1-2x-TAP::kanMX6 exo2-13x-myc::natMX6 h?	This work
exo2-GFP-HA-kanMX6 ade6-216 leu1-32 lys1-131 ura4-D18 h90	National BioResource Project (NBRP), Osaka, Japan
exo2-GFP-HA::kanMX6 h90	This work
eti1-4x-mCherry::hygMX6 ura4-D18 h?	This work
eti1-4x-mCherry::hygMX6 exo2-GFP-HA::kanMX6 h?	This work
eti1-4x-mCherry::hygMX6 exo2Δ::ura4+ ura4-D18 h90	This work
exo2-GFP-HA::kanMX6 eti1Δ::natMX6 h90	This work
exo2Δ::ura4+ ura4- leu1-32 ade6-M216 h-	G. Smith (Seattle, USA)
pabp-eCFP::natMX6 ura4-D18 h90	This work
dcp2-eCFP:: natMX6 ura4-D18 h90	This work
exo2-eCFP:: natMX6 ura4-D18 h90	This work
dcp2-eCFP:: natMX6 ura4-D18 h90	This work
pabp-eCFP::natMX6 exo2-2xGFP::kanMX6 ura4-D18 h90	This work
dcp2-eCFP::natMX6 exo2-2xGFP::kanMX6 ura4-D18 h90	This work
eti1-4xmCherry::hygMX6 dcp2-eCFP::natMX6 ura4-D18 h90	This work
pabp-2x GFP:: kanMX6 eti1-4xmCherry::HygR ura4-D18 h90	This work
pabp-2x GFP:: kanMX6 eti1-4xmCherry::HygR exo2Δ::ura4+ ura4-D18 h90-	This work
dcp2-eCFP::natMX6 eti1-4xmCherry::hygMX6 exo2Δ::ura4+ ura4-D18 h90	This work
dcp2-Tomato::KanR Pabp-eCFP::natR h?	This work
cip2-2xGFP::kanMX6 h90	This work
cip2-2xGFP::kanMX6 sty1-13x-myc::natMX6 h?	This work
sty1-13x-myc::natMX6 h-	This work
pabp-2x GFP:: kanMX6 eti1Δ::natMX6 ura4-D18 h90	This work

Table S1: Strains used in this work

eti1_F	GTATACAGAAGTCGAAGCACGAATTTTTAATTACGACGCTCCAAGCACTGTTAAGAGAAGTCGATATTTTTCTC AATACACGGATCCCCGGGTTAATTAA
eti1_R	TTATAGTTAATAGCTTAAATGCCCCCTATAAGGAAAAAATGCCGTAAAAGACAAAATTACTAGTGCTTGCAGCT AAGCTTGAATTCGAGCTCGTTTAAAC
cip2_F	GTAGTGCGATGAACGATTATTTTGCAAGCCTTACTCCATCAAACACCGGTGCTATTGGATCCAGAACTTTTACA AAGAATCGGATCCCCGGGTTAATTAA
cip2_R	GTACAAAGTATAAATTTGCTTGAAAGCGCTTATTATCGTAAGGTAAATGAAACTTAATAAAACCCGAGACATAG CCAAAAGAATTCGAGCTCGTTTAAAC
exo2_F	GTGGTAAACCCTTAAGGTCGAAAGAACTCAATCGCAAGCACGATCACATCGTTCAGCCGATGGGCAAACTTCAG ATTAACCGGATCCCCGGGTTAATTAA
exo2_R	CTACTAAAATATTTGCACATAAATTTCATTCATATCATAATGTTGTCTTAAAACCTTCTATAAAGTTAAAGAAT TCTATGGAATTCGAGCTCGTTTAAAC

Table S2: Oligonucleotides used in this work
